# Gastric Cancer in the Era of Immune Checkpoint Blockade

**DOI:** 10.1155/2019/1079710

**Published:** 2019-09-24

**Authors:** Lucía Figueroa-Protti, Rebeca Soto-Molinari, Melany Calderón-Osorno, Javier Mora, Warner Alpízar-Alpízar

**Affiliations:** ^1^Research Center in Microscopic Structures (CIEMIC), University of Costa Rica, San José 2060, Costa Rica; ^2^Department of Parasitology, Faculty of Microbiology, University of Costa Rica, San José 2060, Costa Rica; ^3^Cancer Epidemiology Research Program (INISA), Health Research Institute, University of Costa Rica, San José 2060, Costa Rica; ^4^Research Center in Tropical Diseases (CIET), University of Costa Rica, San José 2060, Costa Rica; ^5^Department of Biochemistry, Faculty of Medicine, University of Costa Rica, San José 2060, Costa Rica

## Abstract

Gastric cancer (GC) is one of the most important malignancies worldwide because of its high incidence and mortality. The very low survival rates are mainly related to late diagnosis and limited treatment options. GC is the final clinical outcome of a stepwise process that starts with a chronic and sustained inflammatory reaction mounted in response to *Helicobacter pylori* infection. The bacterium modulates innate and adaptive immunity presumably as part of the strategies to survive, which favors the creation of an immunosuppressive microenvironment that ultimately facilitates GC progression. T-cell exhaustion, which is characterized by elevated expression of immune checkpoint (IC) proteins, is one of the most salient manifestations of immunosuppressive microenvironments. It has been consistently demonstrated that the tumor-immune microenvironment(TIME)‐exhausted phenotype can be reverted by blocking ICs with monoclonal antibodies. Although these therapies are associated with long-lasting response rates, only a subset of patients derive clinical benefit, which varies according to tumor site. The search for biomarkers to predict the response to IC inhibition is a matter of intense investigation as this may contribute to maximize disease control, reduce side effects, and minimize cost. The approval of pembrolizumab for its use in GC has rocketed immuno-oncology research in this cancer type. In this review, we summarize the current knowledge centered around the immune contexture and recent findings in connection with IC inhibition in GC.

## 1. Introduction

Inflammation is an intrinsic feature of cancer, influencing many processes that take place during tumor development and progression [1–3]. In fact, tumor growth is severely compromised if neoplastic cells are not immersed in an appropriate microenvironment in which neoplastic, immune, and other nonimmune stromal cells coexist [4, 5]. This tumor niche is constantly being reshaped as a result of heterotypic signaling between neoplastic and nonneoplastic cells. Given the relevance of the immune contexture in cancer, we are currently witnessing a change of paradigm in cancer therapy, traditionally focused on cancer cells, with the emergence of therapies centered around the TIME [6, 7]. Immune-checkpoint blockade (ICB) is currently at the lead and profiled as the most promising immunotherapeutic approach in cancer [8–10]; however, despite the very encouraging results in some types of cancer, only a subset of patients obtain clinical benefit from ICB. One of the major challenges is, therefore, the identification of precise and accurate biomarkers to personalize ICB in the clinic. Very likely, predictive biomarkers need to be contextualized to each histology [11].

Infection and chronic inflammation are key players in the pathogenesis of GC. *H. pylori* infection, which is particularly linked to GC of intestinal subtype, the most commonly diagnosed worldwide, triggers chronic and persistent inflammation of the gastric mucosa, characterized by intramural infiltration of inflammatory cells and expression of a vast array of inflammatory mediators [12]. Epstein–Barr virus (EBV) is also associated with the etiology of approximately 10% of the GC cases, especially those located in the proximal stomach [13]. Infiltration of the tumor with CD8+ T cells is a common feature of the EBV+ GC [14]. Environmental and genetic determinants are also implicated in the genesis of this malignancy. Thus, the complex interplay of environment, genetics, infection, and inflammation translates into a very heterogeneous disease at the molecular level [14], which ultimately has an impact in the clinical management of the GC patients.

In 2017, the FDA approved the use of the antiprogrammed cell death protein 1 (PD-1), pembrolizumab, in advanced or recurrent GC expressing programmed cell death 1 ligand 1 (PD-L1) [15]. Even before this, various studies had investigated the expression of the PD1/PD-L1 axis in GC, and several found correlation between PD-L1 expression and clinicopathological parameters, including patient survival [16–21]. Interestingly, some reports indicate that *H. pylori* induces the expression of PD-L1 [22–25]. In addition to PD-L1, several other parameters currently suggested as biomarkers of potential clinical relevance for predicting the response to ICB are being studied in GC. In this review, we provide a summary of the current knowledge centered around the immune contexture and the main findings obtained so far in connection to ICB and predictive biomarkers in GC.

## 2. Epidemiology

GC is one of the most important malignancies worldwide. In 2018, this neoplasm accounted for approximately 1,000,000 new cases and 780,000 deaths globally, which makes it the fifth most commonly diagnosed and the third cause of cancer death [26]. Incidence and mortality rates present substantial variations according to geographic location, with well-defined high- and low-risk areas across the world. More specifically, gastric malignancy is highly incident in Eastern Asia, Eastern Europe, and countries located in the Pacific coast of Latin America; in contrast, incidence rates are generally low in Northern America, Northern Europe, Southern Asia, and Australia [27–29]. Mortality rates also show variations with a very similar geographical pattern [27–29]. Interestingly, incidence and mortality rates are 2-fold higher in men than in women [27].

In the last decades, GC incidence rates are experiencing a steady decline globally [27, 29]. Although the reasons remain obscure, it is speculated that this is at least partially attributed to the concomitant decrease in *H. pylori* prevalence, which is a very well-established factor related to the pathogenesis of GC (discussed below). The decrease, however, is not of the same magnitude in GC of different histological subtypes or anatomical locations [27, 30]. The declining trend is particularly connected to a decrease in the incidence of intestinal subtype, whereas the diffuse subtype remains more or less stable [31, 32]. Similarly, GC of the lower part of the stomach is becoming less common, while the rate of cancer of the gastric cardia is increasing, particularly in high-income countries [27, 30, 32]. Although mortality rates also show a global decline, GC is still one of the most important causes of cancer death [27, 29]. At least in some countries, this downward trend in mortality may be partially connected to the implementation of population-based early-screening programs [33–35]. Nonetheless, the 5-year survival rate remains below 30% in most countries, which is mainly related to the fact that most of the cases are diagnosed at advanced stages, when treatment is likely to fail [36]. Studies in population groups with the same ethnic background but dissimilar access to health care, however, suggest that biological factors could also contribute to explain the mortality and survival of GC [37].

## 3. Histopathology

GC is classified, among other factors, according to histopathological characteristics and anatomic location. The Lauren histological classification system is probably the most used and categorizes gastric adenocarcinomas into three main histologic types: intestinal, diffuse, and mixed [38]. Importantly, Lauren histological subtypes show substantial differences at the epidemiological, pathological, and molecular levels [38–41]. Anatomical location of the malignant lesions is also an important parameter in the classification of GC. Marked epidemiological and etiological differences have been revealed for malignant tumors located in the distal part of the stomach and those of the most proximal region [42–44].

## 4. Pathogenesis

The pathogenesis of GC is complex and multifactorial, and differs substantially depending on the histological and anatomical subtype. GC of intestinal subtype, for instance, is the final clinical outcome of a stepwise process known as the Correa Cascade [45]. It starts with *H. pylori* colonization of the normal gastric mucosa, which in conjunction with environmental insults (i.e., diet and lifestyle) triggers a sustained inflammatory reaction resulting in chronic gastritis that, in some patients, may progress to multifocal atrophic gastritis. A subset of them may develop intestinal metaplasia, dysplasia, and ultimately invasive carcinoma [46]. Much less is known about the pathogenesis of the diffuse subtype of GC [47, 48].

Despite the very well-established role of *H. pylori* in gastric carcinogenesis, most of the infected individuals remain asymptomatic or even develop pathologies not related to GC [49]. This feature actually represents one of the most intriguing paradoxes about this bacterial infection. Bacterial strains exhibiting enhanced molecular virulence that ultimately result in stronger inflammatory response are consistently associated with even higher risk of GC [50–52]. Also, a number of polymorphic variants in genes encoding proinflammatory and anti-inflammatory cytokines that play an important role in the immune response triggered by *H. pylori* are also linked to the GC pathogenesis [53–55]. Thus, it is the combination of bacterial, host, and environmental factors what presumably determines the final clinical outcome.

Although the pathogenesis of the malignant lesions arising in the proximal stomach remains very enigmatic [56], EBV is presumably an important etiological factor for tumors at this particular location, especially those located in the cardia and fundus [13]. EBV-positive tumors constitute around 10% of the cases and, given their very distinctive features, they are actually regarded as a different molecular subtype [13, 14].

## 5. Tumor-Immune Contexture in Gastric Cancer

Immune contexture is recognized as a crucial determinant of cancer [1, 57]. Infiltrating immune cells including macrophages, neutrophils, dendritic cells, and several lineages of T cells are major constituents of the tumor microenvironment, participating in many processes that take place during cancer initiation and growth [2, 5].

**Figure 1:**
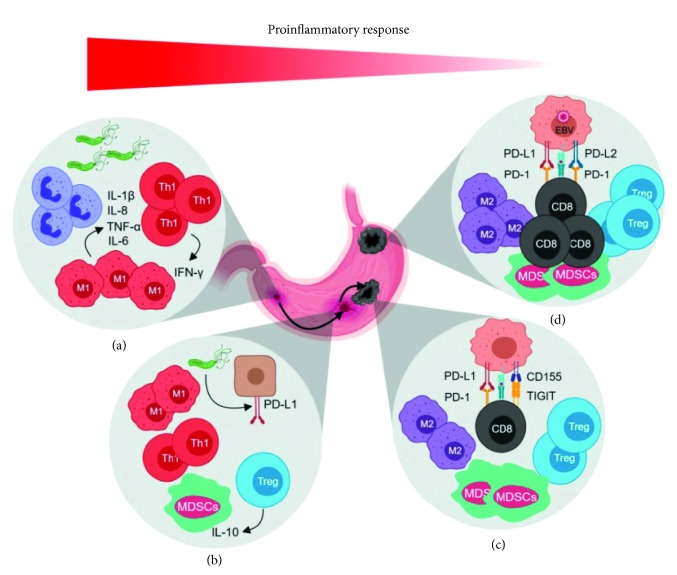
Immune contexture in the pathogenesis of gastric cancer. (a) The colonization of *H. pylori* in the normal gastric mucosa triggers an inflammatory response with accumulation of neutrophils and inflammatory macrophages and production of proinflammatory cytokines such as IL-1*β*, IL-8, IL-6, and TNF-*α*; leading to Th1 polarization, IFN-*γ* production, and chronic gastritis. (b) In later stages, *H. pylori* induces the overexpression of PD-L1 in epithelial cells of the gastric mucosa as an immune evasion mechanism, characterized by an increase in regulatory T cells, myeloid-derived suppressor cells (MDSCs), and IL-10 production. (c) Gastric cancer cells express PD-L1 and CD155 which after interacting with PD-1 and TIGIT on the surface of cytotoxic T cells induce T-cell exhaustion and promote the development of an anti-inflammatory tumor microenvironment. (d) Epstein–Barr virus- (EBV-) positive gastric cancer lesions are mainly located in the proximal stomach and are characterized by amplification and, consequently, high PD-L1 and PD-L2 expression with a prominent immune cell infiltration (created with BioRender.com).

In general, the TIME of overt GC lesions shows an immunosuppressive character (Figure 1(c)). This, however, may vary according to parameters such as tumor histology, anatomical location of the lesion, and molecular subtype, as recently revealed [58]. According to this study, in general, the most prevalent tumor-infiltrating leukocytes were CD8+ T cells, CD68+ macrophages, and CD4+ T cells, representing 15%, 13%, and 11% of all intratumoral cells, respectively. When subdivided according to subtypes, the infiltration with CD8+ T cells, CD4+ T cells, and macrophages was particularly elevated in the EBV+ tumors and the least infiltrated corresponded to the GCs of diffuse subtype. Interestingly, the presence of infiltrating macrophages in GC of intestinal subtype was markedly conspicuous and that of T cells in general was relatively low. Finally, the prevalence of FOXP3+ Tregs in GC was dismal, regardless of the histologic or molecular subtype [58]. Although the latter is probably the most comprehensive, a substantial number of studies have also assessed the composition of the TIME in GC (due to space limitations, we only cite some) [59–69]. Variations in the general prevalence of leukocytes and lymphocytes between studies are expected as a result of the number of patients included, the fact that not all take into account the same clinicopathological and molecular features and the methodological approach used for profiling the immune cell composition and the study design. A general trend, however, in the studies performed so far, is that EBV+ GCs are the most infiltrated tumors, especially by CD8+ T cells. Also, many of the studies have found a significant association between the high number of tumor-infiltrating lymphocytes and improved overall survival, which is particularly robust for the CD8+ T cells [59, 61, 70, 71]. Unlike other types of cancer, there is no current consensus on the morphologic evaluation of tumor-infiltrating lymphocytes in GC, despite some attempts [59]. Therefore, the standardization of a scoring method for quantitation of the tumor-infiltrating lymphocytes in GC lesions is highly needed.

## 6. Immune Exhaustion

T-cell exhaustion was first defined in chronic infections as the failure of effector T cells to acquire a memory T-cell homeostatic state [72]. During an acute infection, a great portion of activated T cells die after the peak of effector expansion; however, a subset persists losing its effector functions and becoming part of the memory T-cell pool. By contrast, in chronic infections, the ongoing antigen stimulation and persistent inflammation induce a progressive loss of effector T-cell functions, but failing to acquire the antigen-independent memory state [72]. One of the most important features of the T-cell exhaustion phenomenon is the progressive increase in the amount and diversity of inhibitory receptors expressed on T cells, including PD-1, cytotoxic T-lymphocyte antigen-4 (CTLA-4), lymphocyte-activation gene 3 protein (LAG-3), T-cell immunoglobulin domain and mucin domain (TIM-3), 2B4, CD160, V-domain Ig suppressor of T-cell activation (VISTA), and T-cell immunoreceptor with immunoglobulin and ITIM domains (TIGIT) [72–74]. Under physiological conditions, these inhibitory receptors, also called ICs, have a crucial role activating negative regulatory pathways in order to prevent autoreactivity and the subsequent immunopathological tissue damage [75]. After T-cell activation, these inhibitory molecules are expressed transiently in functional effector T cells thus maintaining an adequate balance of the immune process [72]. During T-cell exhaustion, however, IC proteins are highly and steadily expressed, and the exhausted phenotype severity depends on the level and number of inhibitory receptors [72].

Although T-cell exhaustion was originally defined in chronic infection, a similar dysfunctional state has been observed in cancer [73]. The role of the immune system in tumor initiation and progression has been widely explored. In fact, the immune-mediated mechanisms play a pivotal role in all stages of tumor biology, regardless of the tissue origin of the tumors. Importantly, the immune system poses a strong selective pressure on the tumor mass that ultimately shapes tumor growth, which has led to the proposal of a cancer-immunoediting process. More specifically, the immune system proceeds sequentially through three distinct phases during tumor development: (1) elimination, in which the innate and adaptive immune systems work together to detect the presence of potentially malignant cells, activate against them, and mediate their destruction; (2) equilibrium, where rare tumor cell variants survive the elimination phase, but the adaptive immune system still prevents their outgrowth and maintains them at bay; (3) escape, in which tumor cells that have acquired the ability to circumvent immune recognition emerge as progressively growing tumors [76]. This last phase can occur through two principal mechanisms: the generation of poorly immunogenic tumor cell variants that are “invisible” to the immune system and/or the establishment of an immunosuppressive state within the tumor microenvironment, which includes the induction of T-cell exhaustion [76]. As a general rule, the inhibitory ligands and receptors that regulate T-cell effector functions in tissues are commonly overexpressed in tumor cells or in nonneoplastic cells in the tumor microenvironment [75].

T-cell exhaustion in cancer and chronic infection share many commonalities, including reduced proinflammatory cytokine production, impaired cytotoxic activity, and elevated levels of multiple inhibitory receptors. Notwithstanding this, differences are also appreciated. In cancer, for instance, priming to tumor antigens is more likely to occur in the absence of inflammation. Consequently, naïve tumor-specific T cells may fail to become properly activated and never differentiate into effector T cells, thus acquiring directly a T-cell exhaustion phenotype [73]. Also, tolerance mechanisms could shape T-cell responses to favor mainly lower-affinity clones [73].

Another important factor influencing intratumoral T-cell activity is the metabolic state of the tumor microenvironment. Effector T cells activate glycolytic pathways for ATP production, even in the presence of oxygen, in a HIF-1*α*-dependent manner [77, 78]. Glucose metabolism in T cells is promoted by HIF-1*α* and the AKT/mTOR pathway, which in turn induces the upregulation of glucose transporter GLUT1, providing the T cells enough energy to perform their effector functions [79]. Tumor cells also reprogram their metabolic pathways towards glycolysis, which is mediated by hypoxia and HIF-1*α*. The fact that proliferating tumor cells increase their glucose uptake limits its availability for T cells as an energy source for their effector functions, affecting the antitumor immune response [80]. Besides changes in the glucose availability in the tumor microenvironment, intrinsic factors in the T cells affect their metabolism. For instance, GC cells express ligands to ICs, such as PD-L1 and CD155, which induce T-cell exhaustion after interacting with PD-1 and TIGIT of the surface of T cells, respectively (Figure 1(c)). PD-1 and TIGIT expression affect T-cell metabolism by inhibiting glycolysis and limiting their effector functions [81, 82]. In fact, downregulation in the expression levels of genes encoding proteins involved in glucose uptake, glucose metabolism, and the AKT/mTOR pathway has been observed in TIGIT+ CD8 T cells from GC patients. Mechanistically, this effect was induced after TIGIT interaction with CD155. Interestingly, the T-cell exhausted phenotype was reversed when the uptake of glucose was increased. Additionally, TIGIT blockade alone or in combination with PD-1 inhibitors improves antitumor immunity in an animal model of GC [82].

As in many other types of cancer, IC overexpression has been described in GC as a mechanism for T-cell exhaustion. Since 2000s, several studies have explored the role of PD-1 and PD-L1 expression in the TIME of GC. More recently, upregulation of other ICs such as CTLA-4, TIM-3, and VISTA has also been reported in human GC [83–85]. Nevertheless, the clinical significance of the differential expression of these immunomodulatory molecules among GC patients has not yet been completely elucidated.

## 7. Immune-Checkpoint Blockade in Gastric Cancer

The description of the T-cell exhaustion phenomenon in the context of cancer and its role in promoting tumor growth led to a paradigm shift in cancer treatment the past decade. The new vision of tumor therapy has focused in the development of approaches that intend to target or manipulate the immune system in order to reactivate antitumor T-cell functions. One of the most significant breakthroughs so far is the pharmacological blockade of PD-1/PD-L1 and CTLA-4 as novel immunotherapeutic options, which reverses T-cell exhaustion and unleashes strong antitumor immune responses. Importantly, the fact that PD-1/PD-L1 inhibition leads to a reduction in tumor load shows that T-cell exhaustion is not a terminally dysfunctional state and that an active and effective antitumor immune response can be restored [73, 75].

The FDA approval of the IC inhibitors pembrolizumab and nivolumab for the treatment of melanoma in 2014 initiated a new era in the treatment of cancer. Since then, a number of PD-1/PD-L1 and CTLA-4 inhibitors have been approved for the treatment of several cancer types, and many clinical trials are currently running [6]. Specifically for GC, the anti-PD-1, pembrolizumab, was approved by FDA in 2017 for its use in advanced, recurrent GC expressing PD-L1, which was based on the phase II KEYNOTE-059 clinical trial [21]. At present, several clinical trials are evaluating other IC inhibitors, including the anti-PD-1 nivolumab, the anti-PD-L1 avelumab, durvalumab, and atezolizumab, and anti-CTLA4 ipilimumab and tremelimumab. In Supplementary [Supplementary-material supplementary-material-1], we summarize the most representative clinical trials evaluating the safety and efficacy of PD-1/PD-L1 inhibitors in GC. Further details of all ICB clinical trials in GC can be found elsewhere [86, 87]. Of note, the phase II trial ONO-4538 and phase III trial ATTRACTION-2 revealed that nivolumab administration to heavily pretreated GC patients is associated with improved overall survival, compared to patients treated with placebo. These results led to the approval of nivolumab in Japan for the treatment of advanced-stage GC patients progressing after standard systemic cytotoxic therapy, regardless of the PD-L1 status [88]. Some of the current trials in GC are evaluating combinations of PD-1/PD-L1 inhibitors with conventional therapies. The MORPHEUS-GC trial, for example, has eight different study groups that combine IC inhibitors, chemotherapeutic agents, MEK inhibitors, anti-VEGF receptor 2 antibodies, PEGylated recombinant human hyaluronidase, CXCR4 antagonists, and DDP-4 inhibitors [86, 87]. Also, the CIRCUIT trail combines ICB therapy with neoadjuvant short-term-limited local radiotherapy [87]. Combinations of IC inhibitors are also being evaluated in GC. The latter is based on previous studies performed in other cancer types showing that combination of two IC blockers leads to significantly improved response rates. In fact, an ongoing phase I/II trial is analyzing the safety and efficacy of nivolumab plus ipilimumab, compared to nivolumab alone, in patients with chemotherapy refractory GC [86, 87].

Other IC proteins are currently studied in preclinical and clinical settings as potential therapeutic targets in cancer, including LAG-3, TIM-3, and TIGIT [6]. In GC patients, for example, TIM-3 and Gal-9 expressions have been associated with poor patient overall survival, suggesting an important role of these molecules in T-cell exhaustion [89]. Furthermore, the potential of LAG-3 as therapeutic target in GC was recently demonstrated in a mouse model using recombinant soluble LAG-3. More specifically, administration of recombinant soluble LAG-3 reduces tumor growth, enhances the secretion of interferon (IFN)-*γ*, promotes CD8+ T-cell activation, and increases the survival rate of GC-bearing mice [90]. In this line, the FRACTION-GC trial seeks to further explore the potential of LAG-3 as a novel therapeutic target by including a group of cancer patients who will receive nivolumab plus an anti-LAG-3 antibody [86, 87].

## 8. Immune-Checkpoint Blockade-Predictive Biomarkers in Gastric Cancer

Cancer patients that respond to ICB generally have long-lasting response rates and manageable safety profile. This, however, is eclipsed by the fact that only a subset of patients derive clinical benefit, which varies according to tumor site. Therefore, the search for biomarkers that can be used in clinical practice to predict the response to ICB is a matter of intense investigation as this may contribute to maximize disease control, reduce side effects, and minimize cost. To date, parameters such as the elevated expression of IC proteins, high mutational load, mismatch repair (MMR) deficiency, microsatellite instability (MSI), high density of infiltrating CD8+ T cells in tumor lesions, and presentation of neoantigens of viral origin are emerging as potential predictive biomarkers [47, 48, 91–96]. Intriguingly, a fraction of patients regarded as potential responders according to these biomarkers do not respond to ICB, which suggest that some parameters of relevance for predicting the response to such agents are still unknown. For instance, studies in melanoma have revealed that the composition and diversity of the gastrointestinal microbiota differ between responders to IC inhibition and nonresponders [97, 98], which is recapitulated in mouse models [99, 100]. An excellent review on ICB predictive biomarkers in cancer has been recently published [101].

A substantial number of studies have characterized the expression pattern of PD-L1 in GC and its correlation with clinicopathological variables. According to these studies, PD-L1 is expressed in 25 to 65% of GC patients, and it is associated with tumor size, lymph node metastasis, and shorter overall survival [18]. Although it has been widely used as companion test in a large number of clinical studies, its utility as a biomarker has been questioned because not all PD-L1+ patients respond to ICB and, even more intriguingly, some negative patients do respond (Supplementary [Supplementary-material supplementary-material-1]). This may be influenced by the lack of a universal cutoff point, differences in the PD-L1 detection assays used, and spatiotemporal intratumor heterogeneity. Many clinical studies using PD-L1 as companion test rely on PD-L1 expression in tumor cells only [8, 9]. More recently, at least in GC, the so-called combined positive score (CPS), which takes into account the PD-L1 positivity on cancer and infiltrating immune cells, has been adopted. This actually showed to be a better scoring method than the percentage of PD-L1+ tumor cells in the KEYNOTE-059 clinical trial with GC patients [70]. Often, clinical trials in GC use a CPS ≥1; nevertheless, it still fails to accurately stratify patients who will benefit from ICB. In the KEYNOTE-061 trial, a CPS ≥10 was evaluated and the overall survival of GC patients treated with pembrolizumab was longer than that of patients under chemotherapy, which could not be recapitulated with a CPS ≥1 (Supplementary [Supplementary-material supplementary-material-1]). These results support the notion that the semiquantitative counting of PD-L1 expression needs to be further refined.

MSI is probably one of the most promising predictive biomarkers for ICB. In fact, FDA approved the use of pembrolizumab in patients with unresectable or metastatic solid tumors with MSI or MMR deficiency, regardless of its tissue of origin. This was actually the first time that FDA approved a cancer treatment based on a common biomarker rather than the location of the tumor. Several of the clinical studies performed in GC have added even more evidence that justifies its use as companion test (Supplementary [Supplementary-material supplementary-material-1]). Interestingly, in the NCT02589496 trial [102], the only GC patient with high MSI that did not respond to pembrolizumab treatment had a heterogeneous MSI pattern, suggesting that it may be relevant to consider the heterogeneity of the tumors when it comes to assessing the MSI status.

In the clinical study by Kim et al. [102], it was revealed that previously treated metastatic GC patients whose tumors were EBV+ respond particularly well to ICB. It has been demonstrated that EBV+ gastric tumors have very distinctive molecular features, including amplification (also overexpression) of PD-L1 and PD-L2, conspicuous intratumoral or peritumoral immune cell infiltration, especially of CD8+ T cells, and IFN-*γ*-driven gene expression profile (GEP) (Figure 1(d)) [14, 103, 104]. These results highlight the potential of EBV positivity as the predictor of the response to IC inhibitors in GC; however, more clinical evidence needs to be added to validate its use in clinical settings. Of note, the studies in GC show that MSI and EBV are mutually exclusive biomarkers [102, 103].

Although tumor-infiltrating lymphocyte density, particularly that of infiltrating CD8+ T cells, is strongly associated with ICB response in several cancer types [9, 48], this has not been rigorously evaluated in GC. Some studies in GC, however, have identified immune-related GEPs that correlate with clinical benefit from ICB therapy, especially the IFN-*γ*-driven and T-cell-inflamed related gene signatures (Supplementary [Supplementary-material supplementary-material-1]). Accordingly, the KEYNOTE-059 trial found that all patients with a PD-L1 CPS >20 had high T-cell-inflamed GEP scores. Elevated expression levels of immune-related gene signatures, however, do not necessarily predict ICB response as this may be influenced by other parameters. This is exemplified in the NCT02589496 clinical trial [102], which revealed that gastric tumors with mesenchymal subtype, defined by elevated expression of an epithelial-to-mesenchymal transition (EMT) gene signature, do not respond to ICB despite exhibiting elevated levels of immune signatures. Indeed, the mesenchymal phenotype has been demonstrated to be a negative predictor of response to ICB in other cancer types and a key determinant of poor survival in GC [105, 106]. According to these results, combination of several parameters may be a better strategy in order to accurately predict the response to ICB in GC.

The use in clinical practice of the approach known as liquid biopsy to determine the response to ICB is highly desirable since this is a noninvasive method that enables the constant follow-up of patients undergoing therapy. With liquid biopsy, for example, it is feasible to determine the tumor mutational load through sequencing analysis of blood-derived circulating tumor DNA (ctDNA). The potential of this approach has been demonstrated in a trial of 69 patients representing 23 different cancer types, concluding that the number of mutations detected in ctDNA was positively associated with ICB response [107]. Similar results were found in a study in non-small cell lung cancer patients treated with atezolizumab [108]. In GC, ctDNA sequencing can reproduce tumor tissue exome sequencing and MSI PCR testing to identify patients likely to respond to pembrolizumab. Even more importantly, posttreatment changes in ctDNA predict both ICB response and progression in GC [102]. These findings expand the possibilities of using liquid biopsy in the clinic to perform evaluations of tumor mutational load as the predictive biomarker of ICB response.

## 9. *Helicobacter pylori* Infection and Its Potential Relevance in the Context of ICB

The inflammatory response mounted against *H. pylori* infection is characterized by a local upregulation in the expression of a vast array of inflammatory mediators and the recruitment of various populations of bone marrow-derived cells to the gastric mucosa, including neutrophils, macrophages, and dendritic, T, and B cells (Figure 1(a)) [109, 110]. *H. pylori* induces the expression of cytokines such as interleukin (IL)-1*β*, tumor necrosis factor-*α* (TNF-*α*), IL-6, IL-8, IFN-*γ*, and the cyclooxygenase (COX)-2 enzyme [111–115], as well as the activation of the transcription factor NF-kB [52, 116]. Most of these effector molecules have pleiotropic effects, thus influencing the progression of *H. pylori*-induced carcinogenesis at different levels. One of the best-studied inflammatory mediators in this context is IL-1*β*, which exerts a proinflammatory function and acts as a strong inhibitor of the gastric acid secretion [112, 117]. The latter creates a less hostile environment for *H. pylori* and other microbial communities lodged in the stomach. In fact, *H. pylori*-colonized individuals with high-expression polymorphisms in the IL-1*β* gene cluster, that is, IL-1*β* and its naturally occurring IL-1 receptor antagonist (IL-1RN), have increased risk for hypochlorhydria, gastric atrophy, and distal GC [53, 118, 119]. IL-1*β per se* is sufficient to induce gastric neoplasia in a mouse model with stomach-specific transgenic overexpression of IL-1*β*, which is mediated by activation of NF-kB and early recruitment of myeloid-derived suppressor cells (MDSCs) to the stomach [117]. Notwithstanding this, the bacterium generally remains in the stomach of colonized individuals for life, indicating that the immune response is ineffective to clear the infection. In addition, the presence of inflammation for decades supports the notion that the immune response is dysregulated by *H. pylori* [110, 120–122]. The mechanisms by which this bacterium modulates innate and adaptive immunity have been reviewed elsewhere [50, 110].

Both *in vitro* and *in vivo* studies have reported that the expression of some IC proteins in gastric epithelial and activated T cells is upregulated upon *H. pylori* colonization, presumably as part of the strategies to evade and subvert host immune defenses (Figure 1(b)). The upregulation of PD-L1 in gastric epithelial cells following *H. pylori* infection is relatively well documented both *in vivo* and *in vitro* [22–25, 123]. Some of the studies addressing this connection have unraveled aspects underlying the induction and regulation of PD-L1 expression in response to *H. pylori*. For example, it has been demonstrated that the induction of PD-L1 in gastric epithelial cells by *H. pylori* requires its type 4 secretion system (T4SS), whose components activate the p38 MAPK pathway [25]. Importantly, studies in mouse models conclude that the upregulation of PD-L1 results in increased bacterial load, induction of Treg cells in the stomach, and increased IL-10 in serum [25]. Elevated expression of PD-L1 in gastric epithelial cells may induce apoptosis of T cells [24]. Also, the induction of PD-L1 expression in GC cells cocultured with *H. pylori* is inhibited by miR-152 and miR-200b [123]. Interestingly, PD-L1 expression is negatively correlated with miR-152 and miR-200b levels in gastric tumor tissues from human patients [123]. The induction of other IC molecules in the context of *H. pylori* infection has also been reported. For example, a recent immunohistochemistry study found higher levels of Gal-3 in the gastric mucosa of patients with *H. pylori* infection, compared to noninfected subjects [124]. Finally, *H. pylori* stimulation resulted in a significant increase of Tim-3 in an *in vitro* system [125]. Altogether, these observations support the notion that IC protein induction in the context of *H. pylori* infection might contribute to the establishment of a persistent infection, which in turn favors the progression from premalignant lesions to gastric adenocarcinoma through the creation of an immunosuppressive microenvironment.

## 10. Conclusions

The establishment of a suppressive TIME is a parameter that greatly influences tumor progression. T-cell exhaustion, through the expression of different ICs, is one of the most salient manifestations of the suppressive TIME. The immune-editing process that takes place during GC initiation and progression, from a proinflammatory state induced by *H. pylori* or EBV infection towards a suppressive microenvironment, includes upregulation in the expression of ICs that prevent T-cell-mediated elimination of tumor cells. The fact that this exhausted state can be reverted with the use of monoclonal antibodies has revolutionized cancer treatment. In the context of GC, the recent approval of the anti-PD-1, pembrolizumab, for the treatment of advanced or recurrent GC represents an important achievement since a large number of patients are diagnosed at advanced stages, when the probability of curing the disease is very limited. A major hurdle, however, is the identification of biomarkers that can be used in the clinic to stratify GC patients and personalize ICB therapy. Until now, there are many promising biomarkers that may be helpful as predictive criteria, but none of them seem to be useful by themselves. Instead, clinical trials reflect the requirement of standardizing an algorithm that includes not one but several of these potential biomarkers, such as PD-L1 expression, microsatellite instability (MSI), MMR deficiency, EBV positivity, immune-related GEPs, ctDNA mutational load scores, and mesenchymal subtype.

The induction of ICs expression in response to *H. pylori* infection is a very fascinating finding that may have important implications in gastric carcinogenesis and, therefore, needs to be further explored. To date, very few studies have addressed the molecular mechanisms underlying this relation. A particularly relevant aspect is whether the induction of ICs in the nonneoplastic gastric epithelium colonized with *H. pylori* has an impact in the composition of the microenvironment of manifest GC lesions. More specifically, it is important to know if the expression of ICs in early stages of carcinogenesis favors the creation of a suppressive inflammatory microenvironment, which facilitates the growth and progression of invasive gastric tumors. High expression of ICs and infiltration by effector T cells from very early stages in the sequence of events that culminates with GC could mean a better response to immunotherapy. Ultimately, all this information may also serve as evidence in favor of the use of ICB therapies in early stages of the disease.

## Supplementary Materials

Supplementary Materials The supplementary material submitted along with the manuscripts is a table in which we aim to summarize the most representative clinical trials for immune-checkpoint blockade therapy in gastric cancer. Most of those studies are ongoing, and a few are already finished. Specifically, in the table, we synthesize the main results obtained in those studies. Of note, in the supplementary table, we only include clinical trials that had a companion test.
