# Supplementary material for: Gastric Cancer in the Era of Immune Checkpoint Blockade
Source: J Oncol. 2019 Sep 24;2019:1079710. doi: 10.1155/2019/1079710 (PMC6778883; doi:10.1155/2019/1079710)
Supplement: Supplementary Materials — The supplementary material submitted along with the manuscripts is a table in which we aim to summarize the most representative clinical trials for immune-checkpoint blockade therapy in gastric cancer. Most of those studies are ongoing, and a few are already finished. Specifically, in the table, we synthesize the main results obtained in those studies. Of note, in the supplementary table, we only include clinical trials that had a companion test. [file 1079710.f1.pdf]

Supplementary Table.

Supplementary Table I. Summary of the most representative clinical trials for immune checkpoint blockade therapy in gastric cancer.

Study* / Regimen	Study population	Biomarker	Main results**	Reference
**KEYNOTE-012 Phase Ib Cohort D** Pembrolizumab	36 patients with advanced PD-L1+ GC	PD-L1 (staining in ≥1% tumor and immune cells)	ORR= 22% (CI 10-39), all PR<br>Patients with tumor size reduction = 53%<br>PFS = 1.9 months (CI 1.8-3.5)<br>OS = 11.4 months (CI 5.7-not reached)	[1]
	33 GC patients: 61% with high Youden index-based cutoff value for the IFN-γ-driven GEP	IFN-γ-driven GEP	IFN-γ-driven GEP associated with a better OR (p<0.10) and PFS (p<0.05).<br>Youden index-based cutoff value for the IFN-γ-driven GEP was necessary, but not always sufficient, for clinical benefit (PPV = 45%, NPV = 92%).	[2]
**KEYNOTE-059 Phase II Cohort 1** Pembrolizumab	259 patients with previously treated advanced GC: 57% PD-L1+	Total cases	ORR in general = 12% (CI 8-16), CR = 2% (CI 1-5)<br>PFS in general = 2.0 months (CI 2.0-2.1)<br>OS in general = 5.6 months (CI 4.3 – 4.3-6.9)	[3]
		PD-L1 (CPS ≥1)	ORR in PD-L1+ patients = 16% (CI 10-22)<br>ORR in PD-L1- patients = 6% (CI 3-13)<br>PFS in PD-L1+ patients = 2.1 months (CI 2.0-2.1)<br>PFS in PD-L1- patients = 2.0 (CI 1.9-2.0)	
	174 patients with previously treated advanced GC: 4% MSI-high	MSI	ORR in MSI-high patients = 57% (CI 18-90)<br>ORR in non-MSI-high patients = 9% (CI 5-14)	
	144 patients with previously treated advanced GC	T-cell-inflamed-GEP	A higher GEP score was associated with improved propensity for response and PFS (p=0.010 and p=0.002, respectively).	

**Study* / Regimen**	**Study population**	**Biomarker**	**Main results****	**Reference**
			All PD-L1 CPS ≥20 had T-cell-inflamed GEP scores in the upper half of the range, but PD-L1 CPS <20 cases were associated with a broad range of T-cell-inflamed GEP scores.	
**KEYNOTE-059**<br>**Phase II**<br>**Cohort 2**<br>Pembrolizumab + Chemotherapy	25 patients with untreated advanced GC: 64% PD-L1+	Total cases	ORR in general = 60% (CI 39-79), CR = 4% (one patient)<br>PFS in general = 6.6 months (CI 5.9-10.6)<br>OS in general = 13.8 months (CI 8.6-not estimable)	[4]
		PD-L1 (CPS ≥1)	ORR in PD-L1+ patients = 69% (CI 41-89)<br>ORR in PD-L1- patients = 38% (CI 8-76)<br>OS in PD-L1+ patients = 11.1 months (CI 5.4-22.3)<br>OS in PD-L1- patients = 19.8 (CI 1.8-not estimable)	
**KEYNOTE-059**<br>**Phase II**<br>**Cohort 3**<br>Pembrolizumab	31 patients with untreated advanced PD-L1+ GC	PD-L1 (CPS ≥1)	ORR = 26% (CI 12-45), CR = 6% (two patients)<br>PFS = 3.3 months (CI 2.0-6.0)<br>OS = 20.7 months (CI 9.2-20.7)	
**KEYNOTE-061**<br>**Phase III**<br>Pembrolizumab vs. Chemotherapy	395 patients with previously treated PD-L1 CPS ≥1 advanced GC	PD-L1 (CPS ≥1)	ORR (Pembrolizumab) = 16% (CI 11-22), CR = 4%<br>ORR (Chemotherapy) = 14% (CI 9-12) , CR = 3%<br>PFS (Pembrolizumab) = 1.5 months (CI 1.4-2.0)<br>PFS (Chemotherapy) = 4.1 months (CI 3.1-4.2)<br>OS (Pembrolizumab) = 9.1 months (CI 6.2-10.7)<br>OS (Chemotherapy) = 8.3 months (CI 17.6-9.0)	[5]
	592 patients with previously treated advanced GC	PD-L1 (CPS ≥10)	Pembrolizumab OS was longer than chemotherapy OS for patients with a PD-L1 CPS ≥10 (HR 0.64, CI 0.41-1.02).	
		MSI	Pembrolizumab OS was greater	

Study[*] / Regimen	Study population	Biomarker	Main results[**]	Reference
			than chemotherapy OS for patients with a MSI-high (HR 0.42, CI 0.13-1.31).	
**CheckMate 032** **Phases I & II** Nivolumab	59 advanced or metastatic esophageal and GC patients: 27% PD-L1+ 12% MSI-high	Total cases	ORR in general = 12% (CI 5-23), CR = 2% (one patient)	[6]
		PD-L1 (CPS ≥1)	ORR in PD-L1+ patients = 19% (CI 4-46) ORR in PD-L1- patients = 12% (CI 2-30)	
		MSI	ORR in MSI-high patients = 29% (CI 4-71) ORR in non-MSI-high patients = 11% (CI 1-35)	
**NCT02589496** **Phase II** Pembrolizumab	61 previously treated metastatic GC patients: 12% MSI-high 10% EBV+ 10% EMT GEP+	Total cases	ORR in general = 25% (CI = 21-46), CR = 5% (CI 1-14)	[7]
		MSI	ORR in patients with MSI-high = 86%, CR = 43% Clinical response was associated with MSI-associated GEP (p<0.0001).	
		EBV	ORR in patients EBV + = 100%, all PR	
		Immune GEP	Clinical response was associated with immune GEP (p=0.007).	
		Proliferation-related GEP	Clinical response was associated with proliferation-related GEP (p=0.030).	
		EMT GEP	ORR in patients EMT GEP + = 0% ORR in patients EMT GEP - = 31%	
	23 previously treated metastatic GC patients	ctDNA sequencing	ctDNA mutational load scores correlated with tumor tissue mutational load scores. ORR in upper tertile of ctDNA mutational load = 83% ORR in two lower tertile of ctDNA mutational load = 8% Change in ctDNA post-treatment predicts response: all patients (n=4) with increasing ctDNA	

Study* / Regimen	Study population	Biomarker	Main results**	Reference
			experienced disease progression within 100 days, all patients with decreasing ctDNA demonstrated significant improvement.	
	55 previously treated metastatic GC patients:<br>51% PD-L1+	PD-L1 (CPS ≥1)	ORR in PD-L1+ patients = 50% (CI)<br>ORR in PD-L1- patients = 0% (CI)	
**JAVELIN Gastric 300 Phase III**<br>Avelumab vs. Chemotherapy	85 previously treated PD-L1+ advanced GC patients	PD-L1	PFS (Avelumab) = 1.4 months<br>PFS (Chemotherapy) = 2.8 months<br>OS (Avelumab) = 4.0 months<br>OS (Chemotherapy) = 4.5 months	[8]
**JAVELIN Solid tumor JPN trial Phase I**<br>Avelumab	40 previously treated advanced GC patients:<br>28% PD-L1+	PD-L1 (staining in ≥1% tumor cells)	ORR in PD-L1+ patients = 27% (CI 6-61)<br>ORR in PD-L1- patients = 4% (CI 0.1-19)<br>PFS in PD-L1+ patients = 1.4 months (CI 0.7-4.0)<br>PFS in PD-L1- patients = 2.6 (CI 1.4-2.8)<br>OS in PD-L1+ patients = 10.9 months (CI 1.0-not estimable)<br>OS in PD-L1- patients = 9.1 (CI 4.9-11.0)	[9]
**ATTRACTION-2 (ONO-4538-12) Phase III**<br>Nivolumab	192 previously treated advanced GC patients:<br>14% PD-L1+	PD-L1 (staining in ≥1% tumor cells)	OS in PD-L1+ patients (Nivolumab) = 5.2 months (CI 2.8-9.4)<br>OS in PD-L1+ patients (Placebo) = 3.8 months (CI 0.8-5.0)<br>OS in PD-L1- patients (Nivolumab) = 6.0 months (CI 4.8-8.5)<br>OS in PD-L1- patients (Placebo) = 4.2 months (CI 3.0-6.9)<br>Exploratory analyses according to PD-L1 positivity in these patients suggest an OS benefit regardless of PD-L1 expression status, with HRs for OS favoring Nivolumab	[10]

Study* / Regimen	Study population	Biomarker	Main results**	Reference
			over Placebo across PD-L1+ and PD-L1+ patient groups.	

CI: 95% confidence interval; CPS: combined positive score; CR: complete response; EBV: Epstein-Barr virus; ; EMT: epithelial-to-mesenchymal transition; GC: gastric cancer (some references include gastro-esophageal junction cancer in this category); GEP: gene expression profile; HR: hazard ratio; IFN-γ: interferon gamma; MSI: microsatellite instability; MSS: microsatellite stable; NPV: negative predictive value; OR: objective response; ORR: objective response rate; OS: overall survival; PD-L1: programmed death-ligand 1; PFS: progression-free survival; PPV: positive predictive value; PR: partial response.

*Only clinical trials that had a companion test were included.

**Central reviewer's criteria reported when available.

Supplementary References

[1] K. Muro, H. C. Chung, V. Shankaran et al., "Pembrolizumab for patients with PD-L1-positive advanced gastric cancer (KEYNOTE-012): a multicentre, open-label, phase 1b trial," *The Lancet Oncology*, vol. 17, no. 6, pp. 717–726, 2016.

[2] M. Ayers, J. Lunceford, M. Nebozhyn et al., "IFN-*c*-related mRNA profile predicts clinical response to PD-1 blockade," *Journal of Clinical Investigation*, vol. 127, no. 8, pp. 2930–2940, 2017.

[3] C. S. Fuchs, T. Doi, R. W. Jang et al., "Safety and efficacy of Pembrolizumab monotherapy in patients with previously treated advanced gastric and gastroesophageal junction cancer: phase 2 clinical KEYNOTE-059 trial," *JAMA Oncology*, vol. 4, no. 5, Article ID e180013, 2018.

[4] Y. J. Bang, Y. K. Kang, D. V. Catenacci et al., "Pembrolizumab alone or in combination with chemotherapy as first-line therapy for patients with advanced gastric or gastroesophageal junction adenocarcinoma: results from the phase ii nonrandomized keynote-059 study," *Gastric Cancer*, vol. 22, no. 4, pp. 828–837, 2019.

[5] K. Shitara, M. ¨ Ozg¨uro˘glu, Y. J. Bang et al., "Pembrolizumab versus paclitaxel for previously treated, advanced gastric or gastro-oesophageal junction cancer (KEYNOTE-061): a randomised, open-label, controlled, phase 3 trial," *The Lancet*, vol. 392, no. 10142, pp. 123–133, 2018.

[6] Y. Y. Janjigian, J. Bendell, E. Calvo et al., "CheckMate-032 study: efficacy and safety of nivolumab and nivolumab plus ipilimumab in patients with metastatic esophagogastric cancer," *Journal of Clinical Oncology*, vol. 36, no. 28, pp. 2836–2844, 2018.

[7] S. T. Kim, R. Cristescu, A. J. Bass et al., "Comprehensive molecular characterization of clinical responses to PD-1 inhibition in metastatic gastric cancer," *Nature Medicine*, vol. 24, no. 9, pp. 1449–1458, 2018.
[8] Y.-J. Bang, E. Y. Ruiz, E. Van Cutsem et al., "Phase III, randomised trial of avelumab versus physician's choice of chemotherapy as third-line treatment of patients with advanced gastric or gastro-oesophageal junction cancer: primary analysis of JAVELIN gastric 300," *Annals of Oncology*, vol. 29, no. 10, pp. 2052–2060, 2018.
[9] T. Doi, S. Iwasa, K. Muro et al., "Phase 1 trial of avelumab (anti-PD-l1) in Japanese patients with advanced solid tumors, including dose expansion in patients with gastric or gastroesophageal junction cancer: the Javelin solid tumor JPN trial," *Gastric Cancer*, vol. 22, no. 4, pp. 817–827, 2019.
[10] Y.-K. Kang, N. Boku, T. Satoh et al., "Nivolumab in patients with advanced gastric or gastro-oesophageal junction cancer refractory to, or intolerant of, at least two previous chemotherapy regimens (ONO-4538-12, ATTRACTION-2): a randomised, double-blind, placebo-controlled, phase 3 trial," *The Lancet*, vol. 390, no. 10111, pp. 2461–2471, 2017.
